# Validation and reliability of Kinovea video analysis for temporomandibular and cervical range of motion in children with spastic cerebral palsy

**DOI:** 10.3389/fbioe.2026.1731269

**Published:** 2026-03-11

**Authors:** Özge Baykan Çopuroğlu, Baki Umut Tuğay, Muhammet Furkan Vatan

**Affiliations:** 1 Department of Therapy and Rehabilitation, Physiotherapy Program, Incesu Ayşe and Saffet Arslan Vocational School of Health Services, Kayseri University, Kayseri, Türkiye; 2 Department of Physiotherapy and Rehabilitation, Muğla Sıtkı Koçman University, Muğla, Türkiye; 3 Department of Dentistry, Dentetik Oral and Dental Health Polyclinic, İstanbul, Türkiye

**Keywords:** cerebral palsy, cervical spine motion, goniometric measurement, Kinovea software, temporomandibular joint, validity and reliability

## Abstract

**Introduction:**

Spastic cerebral palsy (CP) affects mandibular and cervical motor control, making accurate assessment of range of motion (ROM) clinically important. Manual goniometry is widely used but observer-dependent, whereas advanced 3D motion systems are costly. Kinovea software offers a two-dimensional, digital video-based alternative; however, its validity in this population remains unclear.

**Methods:**

Fifty-two children with spastic CP (mean age = 10.8 ± 2.4 years; GMFCS I–IV) underwent mandibular and cervical ROM assessment using manual goniometry and Kinovea video analysis. Two raters performed measurements twice, 1 week apart. Concurrent validity (Pearson’s r), intra/inter-rater reliability (ICC), SEM, MDC95, and Bland–Altman agreement analyses were conducted.

**Results:**

Kinovea demonstrated excellent concurrent validity compared with manual goniometry (r = 0.87–0.99, p < 0.001) across all assessed movements. Intra- and inter-rater reliability were high (ICC = 0.91–0.97). SEM values ranged from 0.41° to 1.01°, and MDC95 values ranged from 1.13° to 2.79°, indicating high measurement precision. Bland–Altman analysis revealed minimal mean bias and narrow limits of agreement, confirming consistent agreement between methods.

**Discussion:**

Kinovea provides precise, reproducible, and clinically feasible measurement of temporomandibular and cervical ROM in children with spastic CP when applied under standardized assessment conditions. The availability of SEM and MDC95 values supports its clinical interpretability for monitoring meaningful changes over time, and its digital nature facilitates integration into routine neurorehabilitation and tele-assessment settings.

## 1 Introduction

Temporomandibular joint (TMJ) dysfunction and restricted cervical mobility are frequent yet often underestimated secondary problems in children with spastic cerebral palsy (CP). Motor impairment in CP is not limited to limb spasticity; it also affects orofacial and cervicothoracic motor control, leading to difficulties in chewing, swallowing, speech, and postural stability (Serel et al., 2022; El Nagar et al., 2023). Limited joint range of motion (ROM) in children with CP has been linked to altered muscle architecture, abnormal neural activation patterns, and secondary musculoskeletal adaptations, which collectively contribute to functional impairment (De Bruin et al., 2013). Altered muscle tone and abnormal synergistic activation in the mandibular and cervical regions result in compensatory movement patterns that further limit functional ROM. In particular, restricted cervical ROM may adversely affect head control, visual orientation, balance reactions, and upper extremity function, thereby influencing overall functional performance and participation in daily activities in children with CP. Quantitative evaluation of these regions is therefore essential to monitor disease severity, optimize rehabilitation strategies, and assess the impact of therapeutic interventions such as manual therapy, neuromuscular re-education, and exercise programs (Scolaro et al., 2022). Moreover, the reliability of ROM assessment is of particular importance in pediatric CP, as measurement inconsistency may directly affect clinical decision-making and longitudinal follow-up (Glanzman et al., 2008). However, despite the clinical importance of orofacial and cervical kinematics, standardized and accessible measurement methods remain limited within pediatric neurorehabilitation practice.

Manual goniometry has long been accepted as the reference standard for joint ROM assessment in clinical settings because of its simplicity and cost-effectiveness. Nevertheless, its precision and reproducibility are strongly influenced by examiner experience, positioning accuracy, and inter-rater variability (Huang et al., 2020). In pediatric CP, involuntary movements and limited cooperation further compromise measurement consistency, highlighting the need for objective, observer-independent tools. In an effort to improve objectivity, previous studies have attempted to assess cervical ROM in children with CP using alternative measurement approaches, including inertial measurement units and photo-based digital techniques (Carmona-Pérez et al., 2020; Johansen et al., 2020). While these methods demonstrated acceptable validity and reliability, their clinical applicability may vary depending on equipment availability, technical expertise, and feasibility in routine rehabilitation settings. Furthermore, recent reviews emphasize the heterogeneity of musculoskeletal and spasticity assessment methods in CP, underlining the ongoing need for standardized and reliable measurement protocols tailored to this population (Nourizadeh et al., 2024).

Advanced three-dimensional motion capture systems provide highly accurate kinematic data, yet their application in children with CP is constrained by high cost, lengthy calibration procedures, and the requirement for specialized laboratory environments (Mazzarella et al., 2020). Consequently, there is a growing interest in two-dimensional (2D) video analysis systems that can deliver clinically acceptable accuracy while maintaining low operational demands. Among these, Kinovea—an open-source video analysis software—has gained increasing attention for its ability to quantify joint motion in rehabilitation and sports sciences (Stenum et al., 2021).

Although Kinovea has been used for analyzing limb and trunk movements in healthy individuals and athletes, its application to orofacial and cervical motion in neurological populations remains largely unexplored. The software allows frame-by-frame angle measurement from recorded videos and provides a digital alternative to traditional goniometry. Several studies have reported strong agreement between Kinovea and laboratory-based motion capture for lower-limb kinematics, with intraclass correlation coefficients (ICC) exceeding 0.90 (Balsalobre-Fernández et al., 2023; Iijima et al., 2023). However, the unique biomechanical characteristics of the TMJ—characterized by coupled rotational and translational components—and the irregular muscle activation patterns observed during cervical motion in children with CP necessitate population-specific validation. Without formal validation against a reference standard, the clinical integration of Kinovea into pediatric TMJ and cervical assessments cannot be justified.

From a methodological standpoint, establishing both concurrent validity and measurement reliability is fundamental when introducing a novel or adapted assessment tool in clinical biomechanics (Koo and Li, 2016). Concurrent validity determines the extent to which a new tool corresponds to a recognized standard, whereas intra- and inter-rater reliability quantify the repeatability of its measurements under consistent and independent conditions. In pediatric neurorehabilitation research, instruments must demonstrate not only statistical significance but also clinical acceptability in terms of minimal detectable change (MDC) and measurement error thresholds (de Vet et al., 2023). Therefore, comprehensive evaluation encompassing validity, test–retest reliability, and inter-rater reproducibility provides the necessary evidence base for translating low-cost video analysis tools into routine clinical practice.

Considering the scarcity of validated digital tools for assessing mandibular and cervical kinematics in children with spastic CP, the present methodological study aimed to examine the concurrent validity and reliability of Kinovea video analysis compared with manual goniometry. By quantifying mandibular and cervical ROM across multiple planes of movement, this research sought to determine whether Kinovea could serve as a clinically feasible, accurate, and reproducible instrument for evaluating joint mobility in pediatric populations with neuromotor impairment. Establishing the psychometric robustness of this approach is expected to facilitate wider implementation of digital motion analysis in both conventional and telerehabilitation contexts, advancing objective outcome measurement within neurorehabilitation biomechanics.

## 2 Methods

### 2.1 Study design and ethical approval

This cross-sectional observational study was conducted between August 2023 and April 2024 at two branches of a pediatric rehabilitation center in Türkiye. The study followed the Standards for Reporting Diagnostic Accuracy Studies (STARD) and Guidelines for Reporting Reliability and Agreement Studies (GRRAS) to ensure methodological transparency. Ethical approval was obtained from the Muğla Sıtkı Koçman University Ethics Committee (Approval No: 98, 24.07.2023). All procedures conformed to the Declaration of Helsinki. Written informed consent was obtained from the legal guardians of all participants, and verbal assent was obtained from the children.

### 2.2 Sample size and participants

The minimum sample size was calculated using G*Power 3.1 software, based on an expected correlation coefficient (r = 0.80) reported in a previous validation study using 2D video-based motion analysis for joint ROM measurement (Harrington et al., 2023). With α = 0.05 and power = 0.90, the required number of participants was ≥45; to compensate for potential dropouts, 52 children were recruited.

The inclusion criteria comprised (1) a diagnosis of spastic CP, (2) age between 7 and 14 years, (3) GMFCS Levels I–IV, and (4) the ability to follow simple verbal instructions and maintain an upright sitting position for at least 10 min, (5) Modified Ashworth Scale (MAS) score ≤2 for cervical flexor/extensor muscles to avoid excessive spasticity affecting cervical kinematics.

Exclusion criteria were designed to minimize confounding and included (1) fixed skeletal deformities of the jaw or cervical spine, (2) previous orthopedic or neurosurgical interventions within 6 months, (3) botulinum toxin or antispastic medication administration within 3 months, (4) acute orofacial pain or temporomandibular joint pathology, and (5) uncontrolled epilepsy or cognitive impairment preventing reliable cooperation.

Diagnosis and GMFCS classification were independently confirmed by a physiotherapist specialized in pediatric neurorehabilitation and a dentist experienced in orofacial evaluation.

### 2.3 Experimental setup and equipment

All procedures were conducted in a standardized clinical environment with controlled lighting and minimal background noise. Three high-definition cameras (Canon EOS 80D, 1080p, 60 fps) were used to capture movement simultaneously from sagittal, frontal, and oblique planes. Each camera was positioned approximately 1.5 m from the participant and aligned at head height (110–130 cm), adjusted individually according to the child’s seated eye level to ensure accurate visualization of temporomandibular and cervical landmarks.

**FIGURE 1:**
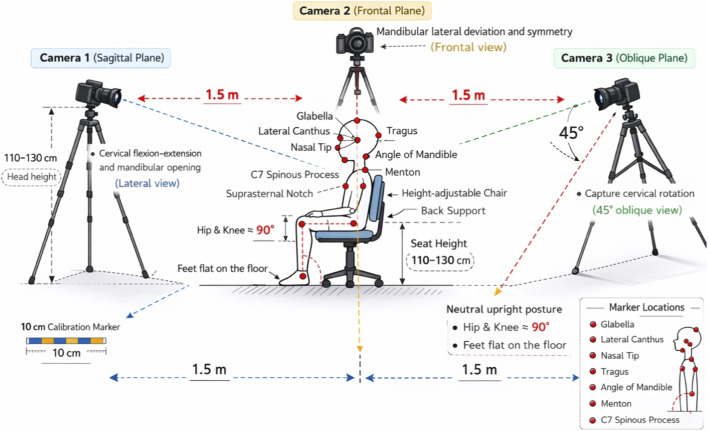
Placement of cameras and markers.

Participants were seated on a height-adjustable chair with back support, positioned to ensure a neutral upright posture. Hip and knee joints were maintained at approximately 90° of flexion, with both feet placed flat on the floor. Trunk alignment was visually monitored to minimize compensatory movements during data acquisition. Camera placement and marker locations are shown in Figure 1.

Camera 1 was placed laterally (sagittal plane) to capture cervical flexion–extension and mandibular opening; Camera 2 was positioned anteriorly (frontal plane) to record mandibular lateral deviation and symmetry; Camera 3 was placed at a 45° oblique angle to accurately capture cervical rotation. This multi-camera setup minimized parallax errors and enabled precise two-dimensional angle extraction.

Manual goniometry served as the clinical reference standard using a stainless-steel universal goniometer (Baseline® 360°, Fabrication Enterprises Inc., USA). For manual goniometric assessment, standardized anatomical landmarks were used, and measurements were performed according to conventional clinical procedures, with the axis aligned over the joint center and the stationary and moving arms aligned with the corresponding anatomical segments. Each movement was measured at the point of maximal active ROM.

Kinovea software (version 0.9.5) was used for digital 2D motion analysis. A 10 cm calibration marker was placed within each camera’s field of view to ensure spatial scaling prior to analysis. All video recordings were synchronized and analyzed frame-by-frame to determine maximal angular displacement.

### 2.4 Marker placement and movement protocol

Each child was seated upright with feet flat on the floor, supported at the trunk to maintain stability. Reflective markers with a diameter of 8 mm were placed on key anatomical landmarks to enhance visibility during video processing. For TMJ evaluation, markers were positioned at the lateral canthus, mandibular angle, and menton for mandibular depression; at the tragus, mandibular angle, and chin for protrusion; and at the glabella, menton, and mandibular angle for lateral deviation.

For cervical motion, markers were placed on the tragus, C7 spinous process, and acromion to measure flexion, extension, and lateral flexion. Although the same anatomical landmarks were used, angular measurements were computed relative to plane-specific reference vectors: in the sagittal plane (tragus–C7 and C7–acromion lines) for flexion–extension, and in the frontal plane (tilt of the tragus–C7 line relative to the acromion or vertical axis) for lateral flexion.

To assess cervical rotation more accurately, additional markers were placed at the nasal tip, tragus, and suprasternal notch. Each movement was performed three times at a comfortable, self-selected speed following standardized verbal instructions. All motions were executed without trunk or shoulder compensation under the supervision of a physiotherapist, and recordings from all three synchronized cameras allowed consistent visualization of the markers in sagittal, frontal, and oblique views.

### 2.5 Measurement procedures

For intra-rater reliability assessment, the same examiner repeated all measurements 1 week later without access to previous data. Inter-rater reliability was evaluated by having a second independent examiner analyze the same video recordings using identical measurement protocols.

**FIGURE 2:**
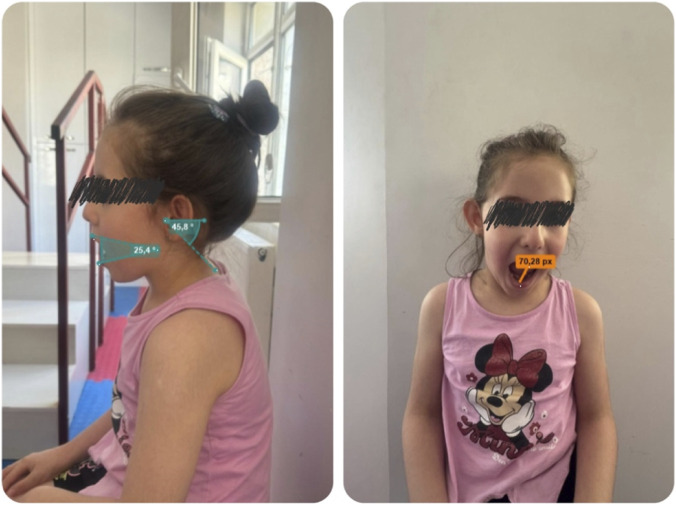
Joint range of motion assessment with Kinovea analysis.

All recorded videos were analyzed frame by frame in Kinovea (Figure 2). The angle tool within the software was used to measure the maximum angular displacement between the designated landmarks during each movement cycle. Calibration was applied using the 10-cm reference marker at the beginning of each analysis to standardize scaling across videos. The three repetitions for each motion were averaged to minimize intra-session variability, and the resulting data were exported to a spreadsheet for statistical analysis.

### 2.6 Outcome measures

The primary outcome variables were mandibular and cervical joint range of motion values obtained using Kinovea video analysis and manual goniometry. Secondary outcome variables included intra-rater and inter-rater reliability indices, the Standard Error of Measurement (SEM), and the Minimal Detectable Change (MDC), which were used to determine measurement precision and clinical interpretability.

### 2.7 Statistical analysis

Statistical analyses were performed using IBM SPSS Statistics v.29.0 (IBM Corp., Armonk, NY) and MedCalc v.22.0 (MedCalc Software Ltd., Ostend, Belgium). Data distribution was verified with the Shapiro–Wilk test, and descriptive statistics were presented as mean ± standard deviation (SD) or frequency and percentage where appropriate. Categorical variables such as sex and GMFCS levels were analyzed using the Chi-square test. The concurrent validity between Kinovea and manual goniometry was tested using Pearson correlation coefficients (r) with corresponding 95% confidence intervals (CI). Agreement between the two measurement methods was further examined using Bland–Altman plots to visualize mean bias and 95% limits of agreement. Intra- and inter-rater reliability were determined by the two-way random-effects intraclass correlation coefficient model (ICC [2,1]) using absolute agreement criteria. Reliability was interpreted as poor (ICC <0.50), moderate (0.50–0.75), good (0.75–0.90), or excellent (>0.90) according to the classification of Koo and Li (2016). The Standard Error of Measurement (SEM) was calculated using the formula SEM = SD × √(1 – ICC), and the Minimal Detectable Change at 95% confidence (MDC95) was computed as MDC95 = SEM × 1.96 × √2. Statistical significance was accepted at p < 0.05.

### 2.8 Quality control and bias prevention

To ensure methodological rigor, several measures were implemented to prevent bias. Both assessors underwent a calibration and training session before data collection, ensuring standardized use of both goniometric and video-based measurement techniques. The order of video analysis was randomized and anonymized by an independent researcher who was not involved in data collection. All recordings were coded numerically to maintain examiner blinding. Equipment calibration was verified before each session, and the environment—lighting, camera position, and participant posture—was maintained consistently across all sessions. Two randomly selected participants were remeasured midway through the data collection period to confirm inter-session reliability and detect any potential drift in measurement accuracy. These procedural controls ensured that the obtained validity and reliability coefficients accurately represented the true measurement characteristics of Kinovea video analysis in the target population.

## 3 Results

A total of fifty-two children with spastic cerebral palsy (28 boys and 24 girls; mean age = 10.8 ± 2.4 years) completed the study without adverse events or attrition. Participants were classified according to the Gross Motor Function Classification System (GMFCS) as Level I (n = 7, 13.5%), Level II (n = 21, 40.4%), Level III (n = 18, 34.6%) and Level IV (n = 6, 11.5%). Descriptive demographic and baseline goniometric ROM findings are shown in Table 1.

**TABLE 1:** Demographic characteristics and baseline goniometric ROM values of participants.

Variable	Mean ± SD or n (%)
Age (years)	10.8 ± 2.4
Sex (Male/Female)	28 (53.8%)/24 (46.2%)
GMFCS level I	7 (13.5%)
GMFCS level II	21 (40.4%)
GMFCS level III	18 (34.6%)
GMFCS level IV	6 (11.5%)
Mandibular depression (mm)	37.35 ± 7.27
Mandibular protrusion (mm)	7.62 ± 2.39
Mandibular lateral deviation – Right (mm)	7.42 ± 2.57
Mandibular lateral deviation – Left (mm)	7.23 ± 2.56
Cervical flexion (°)	35.94 ± 5.43
Cervical extension (°)	35.88 ± 5.69
Cervical rotation – Right (°)	44.10 ± 8.46
Cervical rotation – Left (°)	44.56 ± 8.82
Cervical lateral flexion – Right (°)	34.10 ± 6.74
Cervical lateral flexion – Left (°)	34.15 ± 6.81

Baseline assessment confirmed that cervical mobility was evaluated comprehensively across all primary movement planes, including flexion, extension, rotation, and bilateral lateral flexion.

The correlation analysis demonstrated excellent concurrent validity between Kinovea and manual goniometric measurements across all examined motions. Pearson correlation coefficients (r) ranged from 0.87 to 0.99 (p < 0.001). The strongest correlations were observed for mandibular depression (r = 0.98, 95% CI: 0.96–0.99) followed cervical flexion (r = 0.97, 95% CI: 0.94–0.99) and cervical extension (r = 0.95, 95% CI: 0.91–0.98). Cervical lateral flexion demonstrated excellent concurrent validity, with a Pearson correlation coefficient of r = 0.92 (95% CI: 0.86–0.96), which was comparable to the correlation values obtained for cervical flexion, extension, and rotation. Mandibular protrusion also showed strong agreement (r = 0.94, 95% CI: 0.89–0.97), whereas mandibular lateral deviation exhibited slightly lower yet still excellent correlation (r = 0.87, 95% CI: 0.78–0.93). Cervical rotation yielded a high correlation coefficient (r = 0.90, 95% CI: 0.83–0.94), confirming robust agreement between the two measurement methods across multiple planes of motion (Table 2).

**TABLE 2:** Pearson correlation coefficients (r) and Bland–Altman bias values for Kinovea vs. goniometry.

Motion	r (95% CI)	p	Mean bias (°/mm)	95% limits of agreement (°)
Mandibular depression	0.98 (0.96–0.99)	<0.001	+0.42	−2.03 to +2.87
Mandibular protrusion	0.94 (0.89–0.97)	<0.001	+0.37	−2.45 to +2.82
Mandibular lateral deviation	0.87 (0.78–0.93)	<0.001	+0.84	−2.61 to +3.02
Cervical flexion	0.97 (0.94–0.99)	<0.001	+0.22	−1.96 to +2.40
Cervical extension	0.95 (0.91–0.98)	<0.001	−0.11	−2.35 to +2.13
Cervical rotation	0.90 (0.83–0.94)	<0.001	+0.61	−2.71 to +3.03
Cervical lateral flexion	0.92 (0.86–0.96)	<0.001	+0.35	−2.18 to +2.88

**FIGURE 3:**
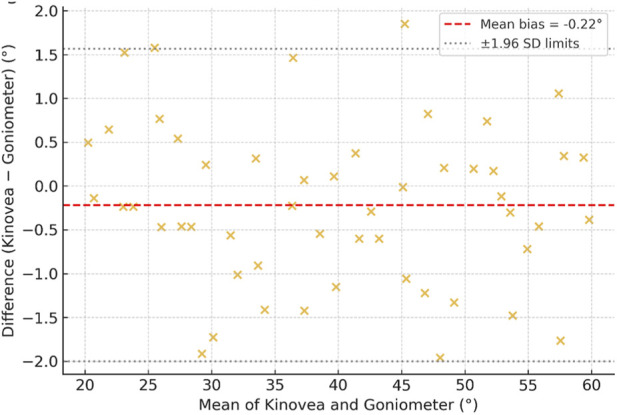
Bland–altman plots comparing Kinovea and goniometric measurements.

The corresponding Bland–Altman plots (Figure 3) revealed mean biases close to zero and narrow 95% limits of agreement, indicating negligible systematic error between the two measurement methods. No trend toward proportional bias was detected across the measurement range, confirming high agreement consistency.

The intra-rater reliability of Kinovea measurements was excellent for all movement directions. Intraclass correlation coefficients (ICC [2,1]) ranged from 0.93 to 0.97, with the highest reliability observed for mandibular depression (ICC = 0.97) and cervical extension (ICC = 0.96). The calculated SEM values ranged between 0.41° and 0.89°, and MDC95 values varied from 1.13° to 2.47°, indicating that even small changes in ROM could be reliably detected in clinical applications (Table 3).

**TABLE 3:** Intra-rater reliability: ICC, SEM, and MDC95 for Kinovea measurements.

Motion	ICC (2,1)	95% CI	SEM (°)	MDC95 (°)	Interpretation
Mandibular depression	0.97	0.95–0.99	0.41	1.13	Excellent
Mandibular protrusion	0.94	0.91–0.97	0.67	1.86	Excellent
Mandibular lateral deviation	0.93	0.89–0.96	0.72	2.00	Excellent
Cervical flexion	0.95	0.92–0.98	0.56	1.55	Excellent
Cervical extension	0.96	0.93–0.98	0.49	1.36	Excellent
Cervical rotation	0.94	0.90–0.97	0.65	1.80	Excellent
Cervical lateral flexion	0.95	0.91–0.98	0.58	1.61	Excellent

ICC (2,1): Two-way random-effects, absolute agreement, single measure.

Overall, the consistently low measurement error indices across all cervical movement directions support the precision and sensitivity of Kinovea for detecting clinically meaningful changes in cervical mobility.

Inter-rater reliability results were similarly robust, with ICC values ranging from 0.91 to 0.95 across all assessed movements. The smallest SEM was observed in cervical flexion (0.54°), and the largest in mandibular protrusion (1.01°), both remaining within clinically acceptable thresholds. The MDC95 values for inter-rater comparison ranged from 1.49° to 2.79°, reinforcing that measurement variability between raters was minimal (Table 4).

**TABLE 4:** Inter-rater reliability: ICC, SEM, and MDC95 for Kinovea measurements.

Motion	ICC (2,1)	95% CI	SEM (°)	MDC95 (°)	Interpretation
Mandibular depression	0.95	0.91–0.97	0.59	1.64	Excellent
Mandibular protrusion	0.91	0.86–0.95	1.01	2.79	Excellent
Mandibular lateral deviation	0.92	0.88–0.96	0.82	2.27	Excellent
Cervical flexion	0.94	0.90–0.97	0.54	1.49	Excellent
Cervical extension	0.93	0.88–0.96	0.73	2.02	Excellent
Cervical rotation	0.95	0.91–0.97	0.62	1.72	Excellent
Cervical lateral flexion	0.94	0.90–0.97	0.69	1.91	Excellent

ICC (2,1): Two-way random-effects, absolute agreement, single measure.

Agreement for cervical lateral flexion between raters was comparable to that observed for flexion, extension, and rotation, further confirming the consistency of lateral cervical motion assessment.

**FIGURE 4:**
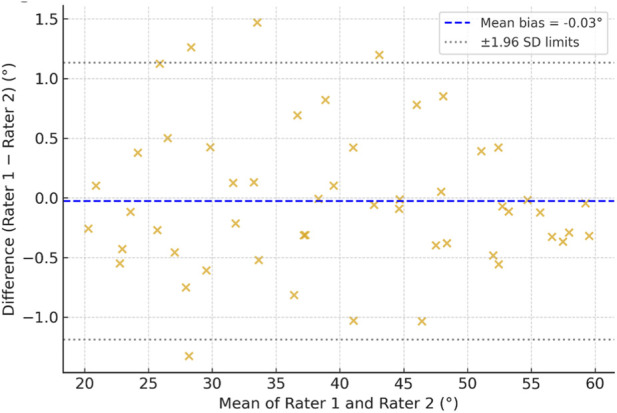
Inter-rater bland–altman plots demonstrating agreement between assessors.

Bland–Altman plots comparing raters (Figure 4) confirmed narrow limits of agreement across all assessed mandibular and cervical movements, with data points symmetrically distributed around the mean bias line, indicating consistent inter-rater agreement and minimal systematic error throughout the measurement range.

The agreement between Kinovea and manual goniometric measurements was further quantified using Bland–Altman statistics. Across all motions, the mean bias ranged from −0.22° to +0.84°, and 95% limits of agreement were within ±2.5°, well below the clinically meaningful threshold for both mandibular and cervical ROM. The absence of significant heteroscedasticity confirmed that the degree of agreement was consistent across the full measurement spectrum. These findings collectively indicate that Kinovea not only correlates strongly with goniometric data but also reproduces its measurements with negligible bias.

Taken together, the results demonstrated that Kinovea video analysis exhibits excellent concurrent validity (r = 0.87–0.99), high intra-rater reliability (ICC = 0.93–0.97), and strong inter-rater reliability (ICC = 0.91–0.95) in assessing both temporomandibular and cervical joint range of motion in children with spastic cerebral palsy. The explicit inclusion and robust measurement performance of cervical lateral flexion, alongside flexion, extension, and rotation, confirm that the assessment protocol provides a complete and multidirectional evaluation of cervical mobility. The low SEM and MDC values confirm the system’s sensitivity to clinically relevant changes, while the minimal mean bias on Bland–Altman analysis supports measurement equivalence between digital and manual methods.

## 4 Discussion

The present findings demonstrate that Kinovea video analysis exhibits excellent concurrent validity and reliability compared with manual goniometric measurement in quantifying temporomandibular and cervical ROM in children with spastic CP. The strong correlations (r = 0.87–0.99) and high intraclass correlation coefficients (ICC = 0.91–0.97) observed across all motion directions confirm that Kinovea can accurately reproduce goniometric measurements while maintaining minimal systematic bias. These results indicate that low-cost 2D video analysis, when applied under controlled conditions, is capable of delivering clinically acceptable precision for evaluating complex motor functions in pediatric neurorehabilitation.

The observed agreement between Kinovea and goniometry supports previous studies that have validated the software for lower-limb and trunk kinematics in various populations (Wochatz et al., 2019; Ore et al., 2020; Vicente-Pina et al., 2025). Similar to those findings, the present results revealed negligible mean bias and narrow 95% limits of agreement, indicating consistent measurement equivalence across the entire motion range. Notably, the degree of correlation found for mandibular and cervical movements exceeds values reported for limb-based assessments in healthy adults (r ≈ 0.85–0.92), suggesting that the structured setup, controlled head stabilization, and marker-based calibration employed here may have minimized parallax and frame distortion errors. This level of methodological standardization is particularly relevant in pediatric CP, where involuntary head and trunk movements can otherwise impair video accuracy (Kim et al., 2018; Doctor et al., 2021; Yan et al., 2024).

The excellent intra- and inter-rater reliability achieved with Kinovea further confirms its repeatability in clinical applications. The ICC values (>0.90) and low Standard Error of Measurement (SEM < 1°) fall well within the thresholds recommended by Koo and Li (2016) for high-stakes clinical decision-making. Importantly, the MDC95 values below 3° provide a clinically interpretable benchmark, indicating that changes exceeding these thresholds are likely to reflect true improvements or deteriorations rather than measurement error. Such sensitivity is critical for monitoring subtle postural or mandibular adaptations resulting from physiotherapy or speech therapy in children with spastic CP. Previous research in adult temporomandibular dysfunction reported inter-rater ICC values ranging from 0.70 to 0.88 using manual goniometry (Marcelino et al., 2024). The higher reliability achieved with digital video analysis in the present context highlights the potential of Kinovea to overcome human measurement error through standardized calibration and automated angle computation.

The methodological strengths of Kinovea stem from its ability to extract angular data from digital recordings while maintaining repeatable reference frames. In contrast to manual goniometry, which relies on single-time-point alignment, video analysis captures the full motion trajectory and allows retrospective review. This feature is particularly advantageous in pediatric populations, where cooperation and movement consistency can vary across trials. The present results align with the emerging consensus that affordable 2D systems, though lacking depth capture, can achieve acceptable validity for functional tasks when proper calibration and plane alignment are ensured (Ota et al., 2020; Agostinelli et al., 2021; Slade et al., 2021). Although the experimental setup required multiple cameras to optimize plane-specific measurements, the overall system remains substantially more accessible and scalable than laboratory-based three-dimensional motion capture systems. Moreover, Kinovea’s open-source nature and ease of integration into telehealth platforms make it well suited for remote rehabilitation monitoring, a need underscored by recent shifts toward digital physiotherapy models (Parks et al., 2019; Armstrong et al., 2022; Kamal et al., 2025).

From a biomechanical standpoint, the strong agreement for both temporomandibular and cervical motions demonstrates that Kinovea can handle small-amplitude, multi-segmental movements with high precision. This finding is important because mandibular motion involves not only rotational but also translational components within the temporomandibular joint, which could theoretically introduce 2D tracking error. However, the camera positioning and reflective marker placement used here appear to have mitigated such artifacts. The consistency observed across multiple movement planes suggests that Kinovea can effectively quantify clinically relevant kinematic parameters even in cases of mild asymmetry or restricted motion. These results expand the utility of Kinovea beyond lower-limb applications and support its adaptation for orofacial and cervical biomechanics research.

The results also carry implications for clinical implementation. Accurate quantification of mandibular and cervical ROM can enhance the assessment of muscle tone, symmetry, and compensatory mechanisms in children with CP. Traditional goniometry remains valuable for quick bedside screening; however, it lacks the ability to provide visual feedback or objective motion replay. Incorporating Kinovea analysis into therapy sessions may improve clinician–patient communication and facilitate outcome tracking over time. Furthermore, the minimal observer bias associated with digital analysis supports its use in multicenter trials and longitudinal studies, where standardization across examiners is essential. The availability of MDC values further strengthens its clinical utility by enabling clinicians to interpret whether observed longitudinal changes exceed measurement error. As clinical practice increasingly adopts digital documentation and tele-rehabilitation systems, the integration of validated tools such as Kinovea can strengthen data quality and reproducibility.

Although the 2D approach used by Kinovea has inherent limitations, such as the absence of depth information and potential parallax effects when out-of-plane motion occurs, these factors were minimized through precise camera alignment and controlled setup. The absence of significant proportional bias in Bland–Altman analysis confirms that such sources of error did not meaningfully affect the results. While three-dimensional motion capture remains the gold standard for high-fidelity biomechanical assessment, its use in pediatric CP populations is limited by cost, setup complexity, and child compliance (Cacioppo et al., 2023; Haberfehlner et al., 2024; Vanmechelen et al., 2024). Accordingly, validated 2D systems such as Kinovea represent a pragmatic alternative rather than a direct replacement for 3D motion analysis, balancing feasibility with acceptable measurement accuracy.

A further strength of this study lies in the comprehensive evaluation of both concurrent validity and reliability within the same experimental framework. Many previous investigations have examined only one of these psychometric dimensions, leading to partial evidence for tool adoption. By incorporating cross-method and repeated-measure analyses, this validation provides a more complete understanding of Kinovea’s measurement behavior in pediatric neurological populations. The use of multiple outcome indices—including ICC, SEM, MDC, and Bland–Altman statistics—ensures that both statistical and clinical aspects of agreement are addressed, thereby satisfying modern standards for biomechanical measurement validation (Ghaderi et al., 2023; Mokkink et al., 2023; Khalifa et al., 2025).

Despite these strengths, certain methodological considerations should be acknowledged. The sample included only ambulant children with GMFCS Levels I–IV, which may limit generalizability to more severely affected individuals. Future research should examine the applicability of Kinovea in non-ambulant or mixed-type CP subgroups, as well as across different age ranges. Additionally, the analysis was performed under static sitting conditions; further studies could extend the validation to dynamic tasks such as chewing, swallowing, or head rotation during functional activities. Exploring integration with wearable inertial sensors or machine-learning–based motion tracking could further enhance precision and automate landmark detection, reducing operator workload.

In conclusion, the findings confirm that Kinovea video analysis provides a valid, reliable, and clinically feasible method for assessing temporomandibular and cervical joint mobility in children with spastic cerebral palsy. Its high correlation with manual goniometry, excellent reproducibility, and minimal measurement bias underscore its potential as an accessible digital alternative for objective kinematic evaluation in pediatric neurorehabilitation. When interpreted alongside MDC thresholds, Kinovea measurements can meaningfully inform clinical decision-making and longitudinal outcome assessment. Adoption of validated open-source tools such as Kinovea can democratize motion analysis, bridging the gap between advanced biomechanical assessment and everyday clinical practice while supporting the evolution of data-driven rehabilitation.

## 5 Conclusion

The findings indicate that Kinovea video analysis provides an accurate, reliable, and clinically feasible alternative to manual goniometry for measuring temporomandibular and cervical range of motion in children with spastic cerebral palsy. The strong concurrent validity and high intra- and inter-rater reliability confirm that the software can deliver reproducible quantitative data with minimal measurement bias across multiple planes of joint motion under standardized assessment conditions.

These outcomes demonstrate that low-cost two-dimensional video analysis, when performed under standardized conditions, can achieve precision comparable to conventional methods while offering the advantages of digital storage, visual feedback, and remote applicability. Importantly, the low standard error of measurement and minimal detectable change values provide clinically interpretable thresholds, supporting the use of Kinovea for monitoring subtle but meaningful motor changes over time in pediatric neurorehabilitation.

The integration of validated open-source systems such as Kinovea into clinical practice may enhance the objectivity of motor assessments, facilitate inter-clinician standardization, and expand access to digital motion analysis as a pragmatic complement to, rather than a replacement for, laboratory-based motion capture systems, particularly in settings where advanced technologies are not readily available. Future research should extend this validation to dynamic functional tasks and explore automated tracking algorithms to further refine accuracy and streamline clinical implementation in both in-person and tele-rehabilitation contexts.

## Data Availability

The raw data supporting the conclusions of this article will be made available by the authors, without undue reservation.
