# Supplementary figures and images for: Validation and reliability of Kinovea video analysis for temporomandibular and cervical range of motion in children with spastic cerebral palsy
Source: Front Bioeng Biotechnol. 2026 Mar 11;14:1731269. doi: 10.3389/fbioe.2026.1731269 (PMC13013515; doi:10.3389/fbioe.2026.1731269)

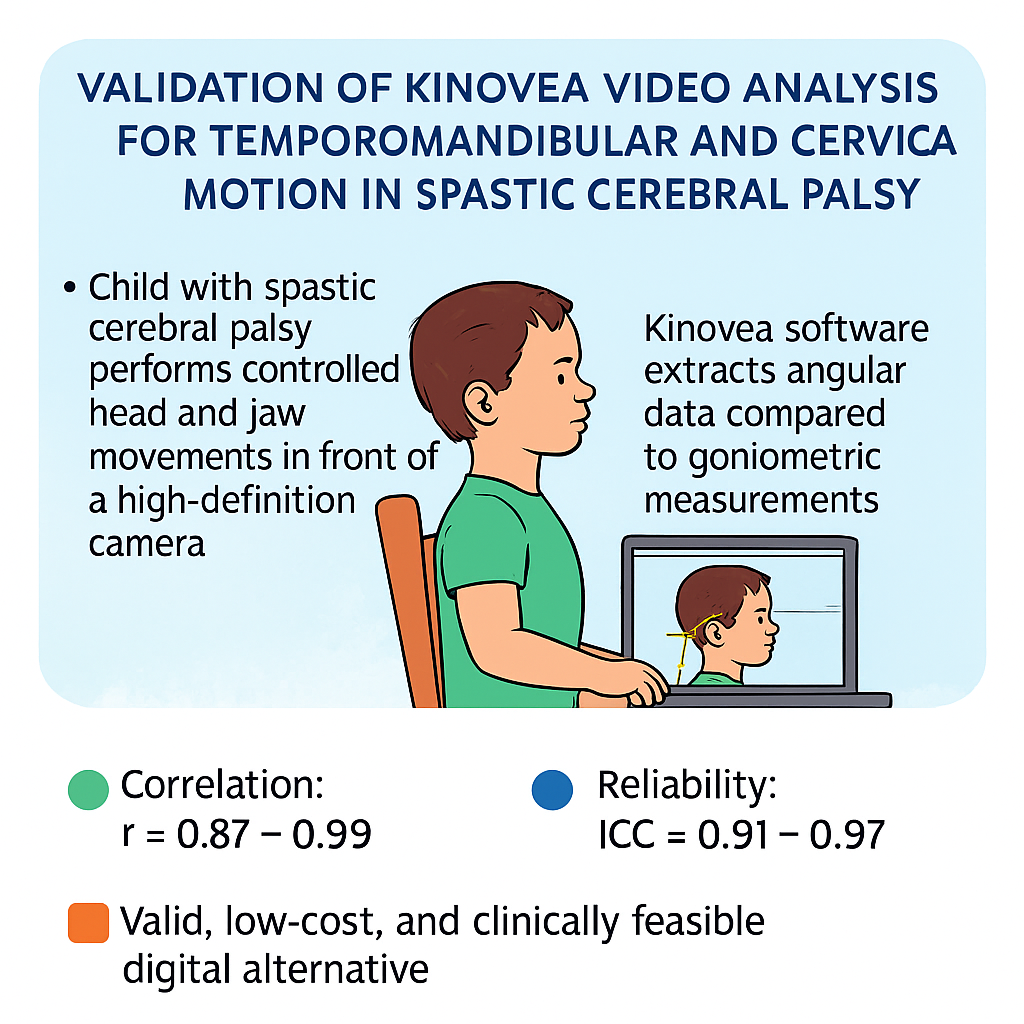

Supplement: Supplementary file 1 [file Image1.png]
